# Supplementary material for: Nineteen-year prognosis in Japanese patients with biopsy-proven nonalcoholic fatty liver disease: Lean versus overweight patients
Source: PLoS One. 2020 Nov 13;15(11):e0241770. doi: 10.1371/journal.pone.0241770 (PMC7665822; doi:10.1371/journal.pone.0241770)
Supplement: S1 File — (DOCX) [file pone.0241770.s001.docx]

**Request for cooperation to a questionnaire survey**

Tokai University School of Medicine

Department of Gastroenterology and Hepatology

Shunji Hirose

This questionnaire has been sent to those who have undergone liver biopsy at Tokai University Hospital since the hospital opened in 1975.

Causes of chronic hepatitis include hepatitis B, hepatitis C, fatty liver, steatohepatitis, alcoholic hepatitis, autoimmune hepatitis (AIH), and primary biliary cholangitis (PBC). However, how each cause affects the prognosis remains unclear.

We are conducting a questionnaire-based clinical study to answer the above question. We sincerely ask you to complete the enclosed questionnaire. Please refer to the alcohol conversion table described below.

We use this questionnaire only for the present research, and we promise to protect personal information. We will consider that you have consented to this research by your responding to the questionnaire survey.

**Please fill out the answer sheet ①. In case that the patient already deceased, please fill out the answer sheet ② on her/his behalf. Finally, please return the answer sheet in the enclosed envelope or fax it to 81-463-93-7134. Thank you very much for your cooperation.**

[Alcohol conversion table] Please refer when answering questions

The following amounts of alcoholic beverages are equivalent to 2 units

Japanese sake 180 ml (1 go)

・Beer 500 ml (a large can of beer, a glass of medium beer mug)

・Canned cocktai**l** (7%) 350 ml (a small can of cocktail)

・Wine (12%) 200 ml (two glass of wine)

・Shochu (25%) 100 ml (half a glass)

・Whiskey, Brandy 60 ml (a double)

143 Shimokasuya, Isehara, Kanagawa. 259-1193

Tokai University School of Medicine

Department of Gastroenterology and Hepatology

**FAX: 0463-93-7134**
