# Supplementary material for: Nineteen-year prognosis in Japanese patients with biopsy-proven nonalcoholic fatty liver disease: Lean versus overweight patients
Source: PLoS One. 2020 Nov 13;15(11):e0241770. doi: 10.1371/journal.pone.0241770 (PMC7665822; doi:10.1371/journal.pone.0241770)
Supplement: S2 File — (DOCX) [file pone.0241770.s002.docx]

**アンケート調査のお願い**

東海大学医学部内科学系消化器内科　広瀬俊治

　このアンケートは1975年の開院から現在までに東海大学病院で肝生検を受けた方に送付させていただいています。

　慢性肝炎の原因にはB型肝炎、C型肝炎、脂肪肝、脂肪性肝炎、アルコール性肝炎、自己免疫性肝炎（AIH）、原発性胆汁性肝硬変（PBC）などがあり、それぞれの原因により肝臓の病気がどのぐらいの速さで進んでいくかはよく分かっていません。

　我々はそのことを明らかにするためアンケート形式の臨床研究を行なっています。つきましては同封のアンケートに御協力をお願いします。尚、飲酒量については下記の換算表を参考にして下さい。不明な部分は空白で構いませんが、可能な限り回答をお願いします。

今回得られた回答内容はこの研究以外に使用することはなく、個人情報は確実に保護されることをお約束します。回答の返送により本研究への同意をいただいたものと判断いたします。

**通常は回答用紙①に、万が一対象者が亡くなっている場合には**

**代理の方が回答用紙②に記入して下さい。回答用紙は同封の封筒で返送、または0463-93-7134までFAXで返送して下さい。**

『アルコール換算表』　アンケートの質問に答える際に参考にして下さい

――　日本酒1合(アルコール約20g)に相当する各種お酒の量　――

・ビール・発泡酒　等 ：約500ml (ロング缶1本、中ジョッキ1杯)

・チューハイ(7度) ：約350ml (ショート缶1本)

・ワイン(12度) ：約200ml (グラス2杯)

・焼酎(25度) ：約100ml (コップ半分)

・ウイスキー、ブランデー ：約60ml (ダブル1杯)

例えば、缶ビール350mlを飲む方は、日本酒0.6合に相当するので、『(○)0～1合』　と記入して下さい。

〒259-1193　神奈川県伊勢原市下糟屋143

東海大学医学部　消化器内科

**FAX　0463-93-7134**
