# Supplementary material for: Nineteen-year prognosis in Japanese patients with biopsy-proven nonalcoholic fatty liver disease: Lean versus overweight patients
Source: PLoS One. 2020 Nov 13;15(11):e0241770. doi: 10.1371/journal.pone.0241770 (PMC7665822; doi:10.1371/journal.pone.0241770)
Supplement: S2 Questionnaire — (DOCX) [file pone.0241770.s004.docx]

**～回答用紙　②～**

(シール貼付欄)

現住所

対象者漢字氏名

通し番号

**生年月日　㍾,㍽,㍼,㍻ ＿＿年　＿＿月　＿＿日**

**記入者 親族/代理者＿＿＿＿＿＿＿＿＿＿（続柄）＿＿＿**

**【問１】 何歳で亡くなられましたか？**

( )　歳

**【問２】 どのような病気で亡くなられましたか？分かる範囲でご記入下さい。**

( )

**【問３】 死亡原因以外に治療をしていた病気はありますか？○をつけて下さい(複数可)。**

(　　　)　脂肪肝 (　　　)　慢性肝炎 (　　　)　B型慢性肝炎 (　　　)　C型慢性肝炎

(　　　)　肝硬変 (　　　)　肝癌

(　　　)　糖尿病(インスリン使用あり) (　　　)　糖尿病(インスリン使用なし)

(　　　)　高脂血症 (　　　)　高尿酸血症 (　　　)　睡眠時無呼吸

(　　　)　高血圧 (　　　)　狭心症 (　　　)　心筋梗塞

(　　　)　脳梗塞 (　　　)　脳出血 (　　　)　認知症

(　　　)　がん　→ どこのがんですか？ ( )

その他の病気がありますか？( )

**【問４】 タバコは吸っていましたか。当てはまるものに○をつけてください。**

(　 )　元々タバコを吸ったことがない

(　 )　吸っていたが禁煙した

→ 何歳まで喫煙していましたか。 ( )　歳まで

→ それまで、1日何本吸っていましたか ( )　本

**【問５】 1日にコーヒーをどれぐらい飲んでいましたか？当てはまるものに○をつけて下さい。**

( )　飲まない ( )　1～3杯 ( )　4杯以上

**【問６】 お酒を飲んでいましたか？当てはまるものに○をつけて下さい。**

( )　全く、あるいは殆ど飲まない　　→　質問は以上です。ありがとうございました。

( )　飲んでいた

**【問７】 何歳からお酒を飲み始めましたか？**

( )　歳

**【問８】 何歳までお酒を飲んでいましたか。**

( )　歳まで

**【問９】 1日平均でどれぐらいお酒を飲んでいましたか？**

**別紙の換算表を参考にして当てはまるものに○をつけて下さい。**

(　　　)　0～1合 (　　　)　1～3合 (　　　)　3～5合 (　　　)　5合以上

**質問は以上です。ご協力ありがとうございました。**
