# Supplementary material for: Nineteen-year prognosis in Japanese patients with biopsy-proven nonalcoholic fatty liver disease: Lean versus overweight patients
Source: PLoS One. 2020 Nov 13;15(11):e0241770. doi: 10.1371/journal.pone.0241770 (PMC7665822; doi:10.1371/journal.pone.0241770)
Supplement: S1 Answer — (DOCX) [file pone.0241770.s005.docx]

**～Answer sheet ①～ Date of birth　 / /**

**Patient ＿＿＿＿＿＿＿＿＿＿＿＿＿＿＿＿**

**Relative/Representative （relationship）**

**【Q1】 Current height and weight**

Height （ ）cm Weight （ ）kg

**【Q2】 Do you smoke?**

(　 )　Never

(　 )　Current　 How many cigarettes a day? （ ）

(　 )　Past

→ How old did you quit smoking? ( ) years old

→ Until then, how many cigarettes a day did you smoke? ( )

**【Q3】 How many cups of coffee do you drink a day?**

( )　None ( )　1 – 3 cups ( )　4 cups or more

**【Q4】 Do you drink alcohol？**

( )　 No　→　**【Q8】**

( )　 Yes, I do. or I drank before.

**【Q5】 How old did you start drinking?**

( ) years old

**【Q6】 Do you drink now？**

( )　Yes

( )　No　→　How old did you quit drinking? ( ) years old

**【Q7】 How many units of alcohol do you usually drink a day?**

(　　) 0-2 units (　　) 2-6 units (　　) 6-10 units (　　　) 10 units or more

**【Q８】 Do you have the following illnesses?**

(　　) Fatty liver (　　) Chronic hepatitis (　　) Hepatitis B

(　　) Hepatitis C (　　) Liver cirrhosis (　　) Hepatocellular carcinoma

(　　) Diabetes (with insulin injection) (　) Diabetes(without insulin injection)

(　　) Dyslipidemia (　　　) Hyperuricemia (　　) Sleep apnea

(　　) Hypertension (　　) Angina pectoris (　　) Myocardial infarction

(　　) Cerebral infarction (　　) Cerebral hemorrhage (　　　) Dementia (　　　) Other cancers→Which organs? ( )

Other illness ( )

**【Q9】 What hospital are you currently visiting?**

（　　　） Tokai University Hospital （　　　） others （ ）

**Thank you for your cooperation.**
