# Supplementary material for: Nineteen-year prognosis in Japanese patients with biopsy-proven nonalcoholic fatty liver disease: Lean versus overweight patients
Source: PLoS One. 2020 Nov 13;15(11):e0241770. doi: 10.1371/journal.pone.0241770 (PMC7665822; doi:10.1371/journal.pone.0241770)
Supplement: S2 Answer — (DOCX) [file pone.0241770.s006.docx]

**～Answer sheet ②～**

**Date of birth　　　 / /**

**Relative/Representative （relationship）**

**【Q1】 Age at death**

( ) years old

**【Q2】 What illness did the patient die from?**

( )

**【Q3】 Did the patient have the following illness?**

(　　　) Fatty liver (　　　) Chronic hepatitis (　　　) Hepatitis B (　　　) Hepatitis C

(　　　) Liver cirrhosis (　　　) Hepatocellular carcinoma

(　　　) Diabetes (with insulin injection) (　　　) Diabetes(without insulin injection)

(　　　) Dyslipidemia (　　　) Hyperuricemia (　　　) Sleep apnea

(　　　) Hypertension (　　　) Angina pectoris (　　) Myocardial infarction

(　　　) Cerebral infarction (　　　) Cerebral hemorrhage

(　　　) Dementia (　　　) Other cancers→Which organs? ( )

Other illness ( )

**【Q4】 Did the patient smoke?**

(　 )　Never

(　 )　Yes

→ How old did the patient quit smoking ( ) years old

→ How many cigarettes did the patient smoke a day? ( )

**【Q5】 How many cups of coffee did the patient drink a day?**

( )　None ( )　1 – 3 cups ( )　4 cups or more

**【Q6】 Did the patient drink alcohol？**

( ) No or very little→　This is the last question. Thank you very much.

( ) Yes

**【Q7】 How old did the patient start drinking?**

( ) years old

**【Q8】 How old did the patient quit drinking?**

( ) years old

**【Q9】** **How many untis of alcohol did the patient usually drink a day?**

(　　　) 0-2 units (　　　) 2-6 units (　　　) 6-10 units (　　　) 10 units or more

**Thank you for your cooperation.**
