# Supplementary figures and images for: Nineteen-year prognosis in Japanese patients with biopsy-proven nonalcoholic fatty liver disease: Lean versus overweight patients
Source: PLoS One. 2020 Nov 13;15(11):e0241770. doi: 10.1371/journal.pone.0241770 (PMC7665822; doi:10.1371/journal.pone.0241770)

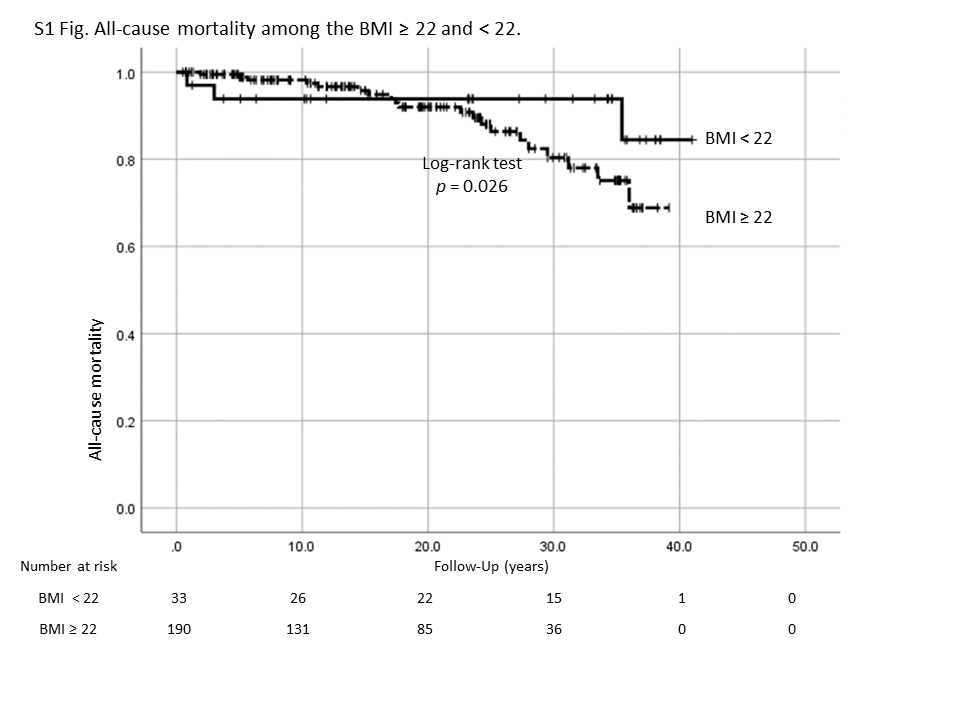

Supplement: S1 Fig — (TIF) [file pone.0241770.s007.tif]

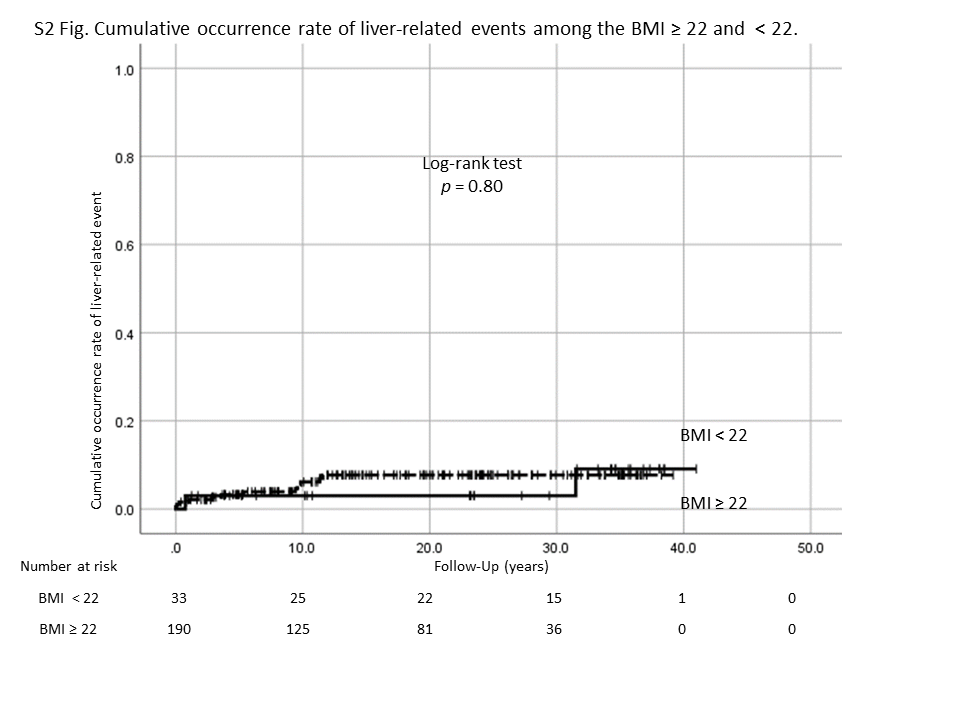

Supplement: S2 Fig — (TIF) [file pone.0241770.s008.tif]
